# The Destruction of the Dental Pulp, and the Treatment of Some of the Diseases Growing out of It

**Published:** 1879-01

**Authors:** Wm. A. Pease

**Affiliations:** Dayton, Ohio


### THE DESTRUCTION OF THE DENTAL PULP, AND THE TREATMENT OF SOME OF THE DISEASES GROWING OUT OF IT.

BY WM. A. PEASE, DAYTON, OHIO.

The sequælæ of the destruction of the dental pulp are often so serious, immediately and remotely, that I approach the consideration of them with hesitation. I look back over the long range of retreating years and see of the numerous cases that have passed before me, not only of my own, but of other's treatment, how few have been wholly satisfactory to patient and creditable to the dentist; and how many have produced blighted hopes and a disappointment of great expectations. Situated on the great highway between the East and the W est, I have had opportunities of seeing numerous cases of cystitis and periostitis after the treatment of distinguished operators, and I have found that there is no *private* highway to success—that failures will come, and failures must be expected. Standing in the immediate presence of such results, I have often wished that the lot might pass from me, and I have looked upon the patient, placidly awaiting the operation, in the expectation of immediate relief, with the thought, alas, he knows not what a day may bring forth.

As a preliminary to the proper development of this subject it is necessary to premise that all pulps can not be destroyed, with reference to filling the teeth, with a just expectation of success. Discrimination must be used, and for a just discrimination there are no universal rules. Diagnosis is individual, begotten on the spur of the moment from accumulated observations, and the impressions one derives from the narration of the experience of others.

In the destruction of the pulp we have chiefly to deal with young persons and with women, for the mature man it is rather exceptional, and the consequences of it are not so serious and intractable. As the great amount of decay commences at or before maturity, the culmination of it in pulp exposure occurs early in life, often in girls before twenty, and before the first gusty flames of puberty have had time to subside. The system is then very impressible, the sexual function active and domineering; it gives an impress to every other aberration of function or pathological change. The condition of the womb being known the prognosis would be comparatively easy, but, as that can only be inferential, we have to call to our aid all the auxiliaries of form, color and expression, which may give a clue to its condition, and which, happily, are not unintelligible. The womb and the ovaries are the presiding genius in the life of women. They give her her buoyancy and spirit; her flushed cheeks and flashing eyes; her pallor and depression, and they make her interesting in the plentitude of her beauty, her moods and her lazy languishment. They indicate the purpose of her being, the intentions of Nature and her position in the scale of life. Their rule is imperative and any infraction of it has its severe penalties, the effects of which are immediate and far-reaching. They mold her whole nature, give her her sympathies, impressibilities, extravagances and tastes, according to their varying conditions and wants.

The more we study them and their surrounding and dependent parts, their nervous connection, immediate or reflex, the more we are persuaded that motherhood was the primal idea of Nature, around which every thing centers and to which every thing tends; and that the want of it is a depravation, and the presence of it is an exaltation of all her faculties and functions and a joy. Then the nerves laugh, the chest expands, the breasts assume a prouder and more imposing mien, the shoulders are not so unseemly obstrusive, and there is a satisfied and contented glow in her expression. But as civilization raises her condition in many respects, it has its corresponding depressors of function and condition; among which are the frequency of miscarriage, often artificially produced, the distractions of society, irregular habits and broken rest; together with inappropriate dressing, which makes the society women not the fittest for an operation or to escape disease, But for these, the destruction of the pulp would not be a forlorn hope, and there would be but little objection to the treatment of the teeth, or the destruction of the pulp for the pregnant or lactating woman, and during the intervals, the indications for treatment ought to be better for her than for her sister of equal age and condition, whose sexual organs have never known the gratification of desire, and through long years have become functionally disordered from quietude and repose. The condition of one is pathological as well as the other, and the hysteria of the latter is due to ungratified sympathies, and the exalted action of some of the nerves at the expense of the others. Her stomach is often impaired; her breast is flattened; her complexion muddy, and there is an anxious expression of face, of longing for a natural gratification that has not yet been attained. The womb and the ovaries may almost be said to be her prima vitæ. Between the two women there can be no doubt but that the one who has been a mother, other things being equal, presents fewer contra-indications for the destruction of the pulp, than she whose maternal instincts have always been in expectancy, and have frequently felt the depressing effects of hope deferred

It may not be desirable to destroy the pulp during the latter months of pregnancy, but if there should be much pain there should be no hesitation about relieving it, and inserting a temporary filling that can be easily removed. As then situated the calls for ossific matter may be greater than her stomach can supply, and there may be a softening of the bones, of which the teeth may give the most noticeable evidence in the exposure of the pulp. This is a pathological condition that by timely observation can be prevented.

Having noticed the two classes of subjects from which the most trouble can be expected, we will now proceed to the destruction of the pulp, to which the paste should be applied immediately from the point of an instrument, and not of cotton, as the contact is better, there is no pressure, and there is less liability to pain. Much of the pain arising from the application comes from pressure of the cap; and a cap containing gum in alcoholic solution is objectionable, as it cuts the creosote, leaving the paste dry, and minus one of the active and soothing ingredients, which in the semi-fluid state secured a closer apposition. There are reasons for leaving the paste in position for twenty-four hours; but where the point of exposure is blind and very small, and the paste can be properly secured, a longer time may be desirable.

After the destruction of the pulp the patient should return in from seven to ten days, generally the latter, to have the operation completed. At that time decomposition has commenced, the attachment to the walls of the tooth has been weakened, and Nature has had time to establish a line of demarkation between the live and dead tissue, generally at the most attenuated point, and it has prepared for the separation without a wound, bleeding or effusion. This is very important, as it supersedes the necessity for any topical application, and if the dam has been adjusted before the pulp is removed no saliva can find entrance, always to be avoided; we can proceed immediately to fill with the greatest prospect of success. But if it is necessary to remove the pulp sooner, there is greater adhesion and more liability to tear it into shreds, a part of which may remain, in spite of every precaution, and there is generally both bleeding and effusion, which we can never be certain, without a too meddlesome interference, of wholly removing. It is of the utmost importance that nothing acrid should go into the canal, nothing but the most simple and non-irritating material, it matters little what it is so it is non-prutrescible, and not a very good conductor. If it is not porous and it is sufficiently soft to adapt itself with moderate pressure to the cavity, so much the better. Different sizes of lead plugs tapering to as near the shape of the cavity as possible, and from one-half to three-fourths of an inch long, possess some advantages rarely equaled by any other material. They are supposed to have a sedative action that is desirable. There now remains that class of cases in which some shreds of the pulp remain adherent to the walls, or there has been effusion or bleeding, each of them a disturbing element, and a source of great uncertainty. Tepid water may remove the soluable parts, but it will not remove the adherent shreds, and it may occasion fresh effusion or bleeding. I can not tell what it may do, and there ought to be no occasion for using it. To swab it out with creosote or carbolic acid would coagulate any effusion or blood, coat the walls of the canal with it, without materially diminishing the quantity, and it would convert it, or the shreds, into a non-prutrescible mass, or at least, prevent it from giving off as much gas as it otherwise would. But there are objections to the introduction of creosote or carbolic acid into the pulp canal, as it might be absorbed by the dentine, and remain and act there for an indefinite period, and, if possible, destroy or diminish the penetration of the fibriles of the peridental membrane, and prevent it from nourishing and sustaining the tooth, as it otherwise would. And besides, the dentine is sparingly soluable in carbolic acid. This may be considered a small matter, but in an operation that demands for success nicety of detail, small matters grow into magnitude. Further objections to the use of creosote or carbolic acid will be made apparent when the treatment of periostitis is under consideration.

The diseases that may follow the destruction of the pulp are three, viz: periostitis. ulceration and necrosis. The first is confined to the connective tissue, the others to the sockets, unless we consider necrosis in that limited sense, that means the death of the tooth from the total destruction of its ministering membrane. Periostitis may arise from various causes as from concussion or catarrh, but it generally arises from blood stasis, or the gas from the decomposition of the pulp, or prutrescible matter in the pulp canal. The two last are the most common, as the destruction of the pulp is not so sudden as to create violent engorgement outside of the pulp canal, and if the case is seen at the commencement, the treatment is both simple and effectual. The periostitis arising from gas is slow, at first a little irritation from a little gas, and scarcely noticeable, this may pass away, but if it does not the volume rapidly increases, and the pressure protrudes the tooth. If the decomposing material is ample, the volume now rapidly increases, setting up an inflammation that may result in caries or necrosis, generally the first, if either, and a chamber is formed in the socket, around the end of the root, generally by pressure, into which it projects. The onset is so sudden, and the vitality is so low, that the destruction is not great or continuous, it subsides after evacuation and a reparatory process is set up. If the periosteum has been simply pealed away from a portion of the root around the foramen, or even if a portion has been destroyed, if the debris of the destructive inflammation does not become adherent to the denuded portion of the root, coating it over, all may yet be well; for the case is so recent that the root has not yet lost the capacity for the periosteum to reform and unite with it. But if the root has been coated that portion becomes absolutely dead, and it must ever after remain a source of irritation to the surrounding parts. At this point the reparatory efforts of Nature may form new bone to surround the denuded and coated portion of the root, but it can not unite with it, and as soon as it grows up to this point it is injured by the protruding root, to again be destroyed by inflammation. The remedy that Nature provided for this was a cyst to surround the end of the root, and by means of its fluid contents to prevent the closure of the cavity and any injury to the soft parts. It is a product of low vitality, of little susceptibility, and yet having sufficient secretory capacity to interpose a fluid to receive and dissipate any shock from sudden concussion or pressure. It intervenes and protects the living membrane of the cavity from injury from the root. It enables the surrounding parts to rest, often in health, or in a sub-acute state of irritation, which does not interfere materially with the usefulness of the tooth. This condition being diagnosed, no treatment is indicated, for nothing can be gained. The sac may be destroyed, but it will then prove a source of irritation until it can be evacuated or absorbed and be succeeded by a new sac.

The idea that the sac is the periosteum detached, pushed away from around the end of the root, and then subsequently enlarged and thickened, that if the tooth were to be extracted, and the sac removed with it, the walls of the cavity would be exposed without a periosteum, like the walls of the pulp canal after the removal of the pulp, is an error. The sac rests against the periosteum of the cavity, and as soon as it is carried away with the tooth, or detached and left behind to be removed by decay, the periosteum commences to fill up the cavity with new bone. Besides the periosteum is not a secretory organ, it could not furnish the fluid that passes through a fistula, and even if it could, and it was a real case of ulceration, the amount of discharge in the course of years would represent a waste that would result in positive deformity, instead of the insignificant cavity there is. A dental cyst not only suffers from mechanical injury, but is exposed to catarrh, and much of the irritation and much of the discharge that flows from it is the result of it. It is catarrh that thickens the fluid and makes it, at times, more offensive than others; but if the discharge does not pass through a filthy tooth, full of all manner of abominations, it is little offensive. The repugnance to it comes from the idea, inculcated, that it is the product of a filthy disease that is dissolving and eating into the bones of the face, and that it is actually dangerous. This causes the loss of many a tooth, cleaner and better than an artificial one, that might otherwise have been useful for years.

From catarrh or injury a low degree of cystitis may arise to give temporary inconvenience, but it is seldom destructive, and teeth in this condition may remain for considerable periods, with short intervals of annoyance, or ought to remind one that disease is present. The advent of a cyst has often been almost imperceptible. A slight and continuous pressure of gas, an absorption of the socket and cavity has slowly increased. The pressure has finally subsided, and a cyst has become necessary to enclose the point of irritation and protect the cavity. Its office is wholly protective, and it is only secretory to give effect to its office. A dental cyst is analagous to a cyst in any other part of the body, modified by the peculiar position and the functions it has to perform. Transferred from the socket, it would soon lose its peculiar characteristics, and be undistinguishable from the cyst that contains a shot.

In connection with a dental cyst there is, occasionally, another condition that merits attention, it is the thickened condition of the periosteum over the whole surface of the root, extending from the junction with the cyst to the edge of the gum. It may have arisen from chronic periostitis, which has gradually pressed the socket away from the sides of the root, and it may have resulted from the cyst having destroyed a part of the support of the root, the tooth is held less firmly and has acquired more lateral motion, a capability of being pressed up farther into the mouth by a full cyst, and of being pressed farther into the cyst during mastication. This would necessitate a greater depth of cyst to prevent contusion of its base, and of the periosteum beneath it, and it would occasion a greater flow through a fistula, the discharge of which may be illustrated by pressure on the rubber bulb of a syringe. In this way, in a healthy subject, a cyst may be so far emptied that it shall cease to discharge; the tooth little used during treatment, it will remain dormant, and the cavity may be carefully filled and everything remain quiet until the socket has had time to contract and hold it more firmly. A small cyst is generally dormant, its functions are passive, and it may exist for years without giving trouble and without an outlet.

These are the conclusions I have been slowly coming to and adopting. About fifteen years ago, while treating some cases that formed the subject of a paper on re-osteogenesis of the dental bones, read before the American Society, at Niagara Falls, the identity of the functions of the dental sac with those of a cyst became apparent to me. I consulted two surgeons of considerable practice, and reading about it, but they did not see it as I did. As time passed on my opinions were strengthened, and two or three times I commenced a paper on the subject, but some point needing further investigation, it was laid aside. Finally a paper was sent to a gentleman, who, probably did not see it as I did, and threw it aside, at least, I heard nothing of it, and suppose it was not used. At the meeting of the society in Philadelphia, in the centennial year, an excellent paper was read by a member in which he likened the sack to a cyst. Who the member was, or how he came to the conclusion I know not, but suppose, as is sometimes the case, that two indedendent persons investigating the same phenomena arrive at the same conclusion. I was glad to have some one think as I did.

There now remains for consideration the treatment of periostitis, by which is ment acute inflammation of the peridental membrane. This membrane is very thin, and it is confined between two bony walls, without much chance for swelling. This makes inflammation of it the more disastrous to it or to the parts which it nourishes. It becomes functionally deranged and sometimes dies, and it occasionally terminates in necrosis or caries. Inflammation of the periosteum is usually the result of the death and decomposition of the pulp; it may arise from other causes, but they are so few and occasional that for all dental purposes these may be assumed to be the cause of it. It is painful and difficult of treatment. Topical applications to the gum are but mitigating; local depletion is little better, and to reach it through the system is tedious and gives it time to be destructive. In the first stage thorough cleaning of the pulp canal is often efficacious and sufficient, but there are cases where the foramen seems to be closed, leaving no direct access, although it is pervious to gas. If a sedative is forced into the canal and confined there, it may be beneficial in time, possibly by imbibation, but the mode of the action is not clearly seen. The introduction of creasote, carbolic acid, iodine, either singly or in conjunction with other materials is empirical and more apt to do harm than good. Bearing in mind the nature of the periosteum and its structure, and having a clean pulp canal, of what benefit can any of them be to a thin, delicate, inflamed membrane in its first inflammatory stage? Directly applied to it, creasote would be escharotic; the action of iodine would be as prompt but more transient, but as its action is to excite the absorbents it will not be of much service here; and tinctures to an inflamed membrane like it are hardly philosophical. Cold applications are better, but their action is not immediate. A prompt purgative, like congress water, cooling topical applications and the expectant theory seem to be the best. This is on the supposition that there is a clean canal. Suppose that there is a part of the pulp, or some other putrescible matter confined beyond access of a removing instrument in the canal—what would be indicated, creosote or the exploring needle? The one might arrest putrefaction, at least in the accessible part; the other would evacuate the gas and relieve pressure. The first would leave in the canal a mass of wholly or partially carbolized material; the other a mass in which putrefaction is exhausted. The one having creosote or some other material in it to act indefinately, perhaps to volatilize and pass through the foramen, the other wholly inert, and neither of them in proximity or at but the minutest point to living tissue. In the one case no chemicals have touched the dentine it is in its natural condition; in the other it has imbibed some of them, and to that extent the worse, leaving out of view the effect they may have on the fibrils of the membrane that penetrate very near to it. In three months after the application extract two teeth treated in this way and a section of the dentine at the place of application, would show very different results; the one would show the effects of the creasote in a darkened bone; the other would be light and healthy. Which would be the best neighbor to the fibrils of the periosteum? It is for their thrift and health we must care.

Thus far I have proceeded on the supposition of acute periostitis. Suppose it is now sub-acute, chronic, with a tendency to ulceration, what is to be done? What can we do? Let us see what is usually done and the effects of it, and perhaps it will furnish a clue to further action. Our old friend, creosote, will still come in play, will it not? I think nearly every one will answer yes. Let us bear in mind the thin, delicate membrane as before, and suppose creosote to be directly applied to it; there is nothing to be disinfected, no putrescence to arrest, because we suppose the canal to be clean. The action of creosote is either anti-putrefactive or escharotic. Let us inject a little through the foramen. It reaches the periosteum, and if we can not see it, a white patch is there the same as there would have been on the lip, or the mucus membrane of the mouth, and all know what that means—a dead surface. But a dead surface spot on the periosteum is perforation, death through it to the bone, and denuded, spongy bone is suggestive of caries. This is before effusion, but if effusion has taken place the fluid should be removed, and the rest and the absence of pressure will generally be sufficient. Leaches to the gum may help, but rest in a healthy constitution is what is wanted. Inflammation of the periosteum may come from the mallet, and it may be temporary or persistent. It may be a patch around the foramen and soon subside, but, like all periosteal diseases it may be very injurious. A general inflammation, embracing the whole surface from the foramen to the gum is apt to localise in a line or strip, frequently on the labial side of the single rooted teeth. The part dies and there is a passage of dead periosteum from th foramen to the edge of the gum, which soon becomes a passage for a discharge. On the multirooted teeth it may be either on the labial or palatine side, but on the molars the large palatine root is generally the one. The continuous discharge irritates the remaining periosteum; it gradually dies and the socket over the part having no periosteum wastes and soon dissappears, leaving the part imperfectly covered with the gum, which soon wastes and there is so much irritation the tooth must be removed.

After the pulp has been destroyed and removed, the canal filled, and we have placed the tooth in the best condition within our present knowledge, the tooth may be uneasy or troublesome. The nerve that presided over its nutrition, that regulated the flux and reflux of blood to its very center, is gone; its nourishment is impaired and its ability to adjust itself to thermal and electric changes. The periosteum has a double task to perform, which undergoes engorgement, but still the tooth may be durable and valuable. When can we learn people, if they wish to preserve their teeth, not to neglect them until the pulp is exposed?

To illustrate the subject better let us have a familiar clinic by way of summary, and if it does not show any new means of treatment it may show what to avoid.

Scene, a dental office abroad.—A gentleman in the chair; the dentist invites me to the chair and introduces me to the patient whose mouth is covered with the dam. The tooth is rather difficult to fill, requiring some building up; it is well prepared; he takes the hot air syring and injects it; the cavity looks white and dry except near the pulp canal; he tries again, the same; he looks puzzled and wraps some cotton on the point of an instrument, presses it into the canal, twirls it around, withdraws and the cotton is wet; he tries again, the same; he is getting nervous, the tooth is behaving badly in company; he again wraps the instrument, dips it in creosote, pushes it home in the canal, twirls it around, withdraws it, and it is coated on the end with a yellowish, curdy matter, that is colored with creosote to look like pus; he tries again with no better result, the cotton being rather more soiled than before; he then informed the sitter that it was an ulcerated tooth that could not be filled at that sitting, but it must be treated; he then saturated more cotton with creosote, pressed it home, left it there, covered it with a cap, removed the dam and dismissed him. The condition of the tooth was this: it had a small cyst that had probably never given him any trouble, of the presence of which the dentist was probably ignorant. The pressing of the instrument armed with the cotton down the canal forced the air by and through it, until it became moist near the foramen, the twirling it around wet it still more, and in withdrawing it, it acted like a pump and drew the effusion from the cyst. The creosote coagulated the very watery effusion and the flaky, cheesy coagula looked like pus. Although there was but little of it, there was enough to show on the cotton and to adhere to and dirty the walls of the cavity with so thick and adherent a material that no syringe could remove. By this time the cyst was probably empty. Now suppose the creosote had been forced into the cyst through the foramen. What would be the result? If there was any effusion it would be coagulated and the coagula could not probably pass through the foramen; the cyst might have been irritated, destroyed, leaving such a mass of carbolized tissue there that would not easily break down minute enough to pass through the foramen or a fistula. Supposing another injection of creosote, there being no living tissue there to receive it, it might have acted upon the periosteum of the bone, and what will it be, necrosis or caries?

Now if that dentist, instead of stopping there had taken the cotton on the end of his instrument for a pattern, selected a lead plug as near to it as possible, and then filed it to fit, pressed it to the bottom of the canal, tapped it gently, it would have been nearly or quite a water tight fit. There could be no change in it, scarcely oxydation, in the absence of air. The tooth filled would probably be in a far better condition than it would be after treatment. The pumping out of the cyst with cotton on the point of an instrument was a good device, although he did not know it. It is what I frequeutly do in incipient periostitis to open the foramen and liberate gas before injecting cold water to reduce temperature, and in catarrhal cystitis to draw the thickened effusion through the foramen and relieve tension.

Another device a little more troublesome, but more effectual in some cases, is a syringe, to the nozzle of which has been fitted a rubber or gutta percha disk about the size of the pulp canal. Two or three sizes of these caps or disks are sufficient. When wanted for use pass one onto the tube a little past the nozzel and apply it to the opening of the canal. If it does not fit air tight try another, or cut a piece from the rubber dam a little larger, cut a very small hole in the center, press it over the nozzle and it will be air tight. Place it in position, caution the patient to sit still and not to grab it, and begin to slowly withdraw the piston. It will withdraw the air and gas from the canal and from beyond the foramen. For the thickened effusion of catarrhal or chronic cystitis, the pumping must be very slow and long continued, as it is but semi-fluid, moves slowly and with difficulty passes the foramen. The same kind of disks are useful in injecting the canal or a fistula, but it is necessary in order to fill them to have a very fine hole through them to allow the compressed air to escape, but not large enough for the water. They are handy to have near, but the pumping is often painful.

We can not all of us think or see alike; therein lies the road to progress.

